# Native American ancestry and breast cancer risk in Colombian and Mexican women: ruling out potential confounding through ancestry-informative markers

**DOI:** 10.1186/s13058-023-01713-5

**Published:** 2023-10-02

**Authors:** Linda Zollner, Diana Torres, Ignacio Briceno, Michael Gilbert, Gabriela Torres-Mejía, Joe Dennis, Manjeet K. Bolla, Qin Wang, Ute Hamann, Justo Lorenzo Bermejo

**Affiliations:** 1 Statistical Genetics Research Group, Institute of Medical Biometry, Heidelberg University, Heidelberg, Germany; 2 Molecular Genetics of Breast Cancer, German Cancer Research Center (DKFZ), Im Neuenheimer Feld 580, 69120 Heidelberg, Germany; 3 Division of Proteomics of Stem Cells and Cancer, German Cancer Research Center (DKFZ), Heidelberg, Germany; 4 Institute of Human Genetics, Pontificia Universidad Javeriana, Bogotá, Colombia; 5 Instituto de Genética Humana, Universidad de la Sabana, Bogotá, Colombia; 6 Center for Population Health Research, National Institute of Public Health, Cuernavaca, Morelos Mexico; 7 Centre for Cancer Genetic Epidemiology, Department of Public Health and Primary Care, University of Cambridge, Cambridge, UK; 8 Department of Biostatistics for Precision Oncology, Institut de Cancérologie Strasbourg Europe, Strasbourg, France

**Keywords:** Genetic admixture, Ancestry-informative markers, Causal inference, Instrumental variables, Mendelian randomization

## Abstract

**Background:**

Latin American and Hispanic women are less likely to develop breast cancer (BC) than women of European descent. Observational studies have found an inverse relationship between the individual proportion of Native American ancestry and BC risk. Here, we use ancestry-informative markers to rule out potential confounding of this relationship, estimating the confounder-free effect of Native American ancestry on BC risk.

**Methods and study population:**

We used the informativeness for assignment measure to select robust instrumental variables for the individual proportion of Native American ancestry. We then conducted separate Mendelian randomization (MR) analyses based on 1401 Colombian women, most of them from the central Andean regions of Cundinamarca and Huila, and 1366 Mexican women from Mexico City, Monterrey and Veracruz, supplemented by sensitivity and stratified analyses.

**Results:**

The proportion of Colombian Native American ancestry showed a putatively causal protective effect on BC risk (inverse variance-weighted odds ratio [OR] = 0.974 per 1% increase in ancestry proportion, 95% confidence interval [CI] 0.970–0.978, *p* = 3.1 × 10^–40^). The corresponding OR for Mexican Native American ancestry was 0.988 (95% CI 0.987–0.990, *p* = 1.4 × 10^–44^). Stratified analyses revealed a stronger association between Native American ancestry and familial BC (Colombian women: OR = 0.958, 95% CI 0.952–0.964; Mexican women: OR = 0.973, 95% CI 0.969–0.978), and stronger protective effects on oestrogen receptor (ER)-positive BC than on ER-negative and triple-negative BC.

**Conclusions:**

The present results point to an unconfounded protective effect of Native American ancestry on BC risk in both Colombian and Mexican women which appears to be stronger for familial and ER-positive BC. These findings provide a rationale for personalised prevention programmes that take genetic ancestry into account, as well as for future admixture mapping studies.

**Supplementary Information:**

The online version contains supplementary material available at 10.1186/s13058-023-01713-5.

## Introduction

Breast cancer (BC) is a major public health concern, as it is the most commonly diagnosed cancer among women worldwide and the leading cause of cancer death [1]. BC is a complex trait with multiple established genetic and non-genetic, risk and protective factors influencing disease development.

Latin American women and Latinas are ethnically diverse [2–8]. Their genomes are made up of chromosomal segments of Native American, European and African origin admixed in different proportions within and between countries of South America, the Caribbean, and Mexico, depending on the original peoples and the specific local history of European settlement and the slave trade [9–16]. In Mexico, individual Native American proportions are generally highest in the center and south of the country, European percentages are highest in the north, and the African contribution to the Mexican genome is generally limited, with the exception of some coastal regions. In Colombia, Native American proportions are particularly high in the east and southwest of the country, central areas show generally high percentages of European ancestry, and proportions of African ancestry increase in coastal regions, especially along the Pacific [17].

Differences in the risk of various diseases, including different types of cancer, according to the individual proportion of Native American, European and African genetic ancestry have recently moved into focus [18, 19]. For example, prostate cancer is more common in black men than in white men, and genome-wide association studies have identified variants at 8q24 that account for a large proportion of the excess risk of prostate cancer in black men [20]. The relative proportion of European versus Native American ancestry has been associated with BC risk in US Latinas and in Mexican and Colombian women. Women with a high proportion of Native American ancestry have a lower risk of BC than those with a high proportion of European ancestry [21–25]. The reported protective effects of Native American ancestry take into account statistically established risk factors such as oral contraceptive use, parity, age at first full-term pregnancy, age at menarche and breastfeeding, but at least part of the association could be attributed to as yet unknown confounding factors, which may be specific to Latin American and Hispanic women, and to measurement errors in the adjustment variables (e.g., accurate information on socio-economic status is difficult to obtain). The origin of the negative association between Native American ancestry and BC risk—are confounding factors, e.g. higher parity or lower income of women with a high percentage of Native American ancestry, responsible for this association?—has important implications for BC prevention and research.

Genetic variants (e.g. single-nucleotide polymorphisms [SNPs]) can be used as instrumental variables (IVs) to assess the confounding-free effect of an exposure of interest (in this study, the individual proportion of Native American ancestry) on a given outcome (in the present study, BC development) [26]. The alleles of the SNPs used as instruments for the proportion of Native American ancestry are randomly allocated during meiosis, mimicking a trial randomized by nature and resulting in effect estimates that are less influenced by potential confounders than those from observational studies [27]. Instrumental variable analyses on the relationship between the individual proportion of Native American ancestry and BC risk would facilitate the distinction between correlation and confounder-free association, allowing prioritisation of unconfounded associations for more efficient prevention and providing the basis for further analysis to better understand the non-genetic and genetic mechanisms leading to BC development.

With these objectives in mind, we preselected ancestry-informative markers (AIMs) in reference panels of Native American, European and African ancestry using the informativeness for assignment measure [28]. We then selected valid IVs in Colombian and Mexican women separately that were robustly associated with the proportion of Native American ancestry (relevance assumption), not associated with potential confounders (independence assumption) and not directly associated with outcome (exclusion restriction assumption). Next, we performed two-sample Mendelian randomization (MR) analyses, which we complemented with sensitivity analyses and analyses stratified by age at diagnosis, family history and BC hormone receptor status. Population stratification, usually a limitation in genetic association studies, was our exposure of interest, with an atypically high proportion of variance explained by the IVs used (between 7 and 39%).

## Results

**Fig. 1:**
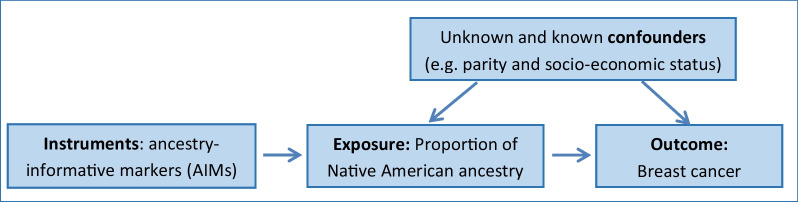
Typical directed acyclic graph of Mendelian randomization adapted to the present study

Figure 1 depicts the usual directed acyclic graph of MR adapted to the present study. Increasing individual proportions of Native American ancestry have been linked to decreasing BC risk, but this association may be attributed to some degree to unknown confounders and non-random measurement error in known confounders (e.g. socio-economic status), motivating the use of AIMs as instrumental variables to assess the unconfounded effect of the proportion of Native American ancestry on BC risk.

**Fig. 2:**
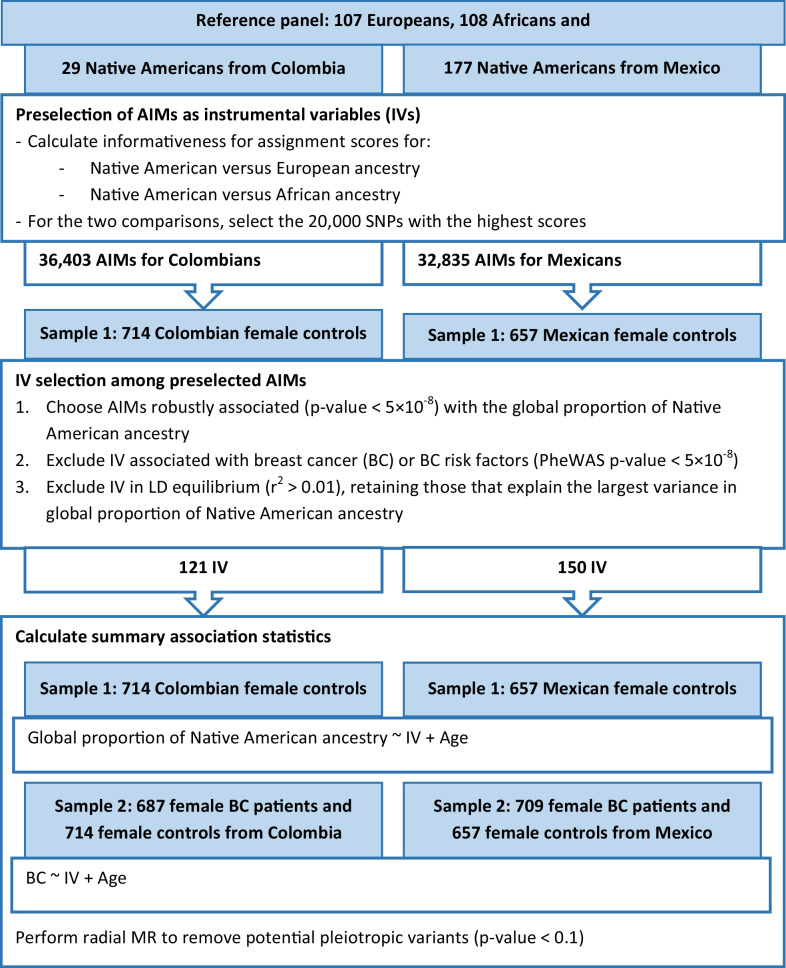
Flowchart representing the statistical analyses for Colombian and Mexican study participants from the preselection of markers of Native American ancestry to the two-sample Mendelian randomization (MR) analysis

Figure 2 represents the analyses conducted from AIM pre-selection to the two-sample MR analyses. The left part of the flowchart shows some intermediate results based on Colombian women, while the right part refers to the analyses in Mexican women. The ancestry reference panels in the primary analyses included 107 Iberians from Spain (IBS) and 108 Yorubans from Ibadan, Nigeria (YRI) from the 1000 Genomes Project, complemented with 29 Native Americans from Colombia (nine Inga, five Arhuaco, four Embera, three Kogi and three Waunana described by Reich et al. and five Piapoco individuals from the Human Genome Diversity Project [HGDP]) or 177 Native Americans from Mexico (49 Maya, 43 Zapotec, 30 Pima, 25 Tepehuano, 17 Mixe, five Mixtec, one Yaqui and one Purepecha described by Reich et al. and four Maya and one Pima from the HGDP) [29, 30]. Relying on the informativeness for assignment measure, we preselected 36,403 AIMs for Colombian women and 32,835 AIMs for Mexican women. Among the preselected markers, 623 were robustly associated (*p* < 5 × 10^–8^) with the proportion of Native American ancestry among Colombian female controls; the corresponding number in Mexican female controls was 6118. Downstream phenome-wide association analyses (PheWAS, using a *p*-value threshold of 5 × 10^–8^) and linkage disequilibrium (LD) pruning (*r*^2^ > 0.01) resulted in 121 preliminary IVs for the subsequent Colombian analyses and 150 IVs for the subsequent Mexican analyses. We then complemented age-adjusted summary statistics on the association between the IVs and the proportion of Native American ancestry with age-adjusted summary statistics on the association between the IVs and BC risk based on BC patients and controls to perform radial MR and exclude potentially outlying instruments (*p* < 0.1). Finally, 98 and 134 IVs were utilised to investigate the confounder-free effect of the proportions of Colombian and Mexican Native American ancestry on BC risk.

**Fig. 3:**
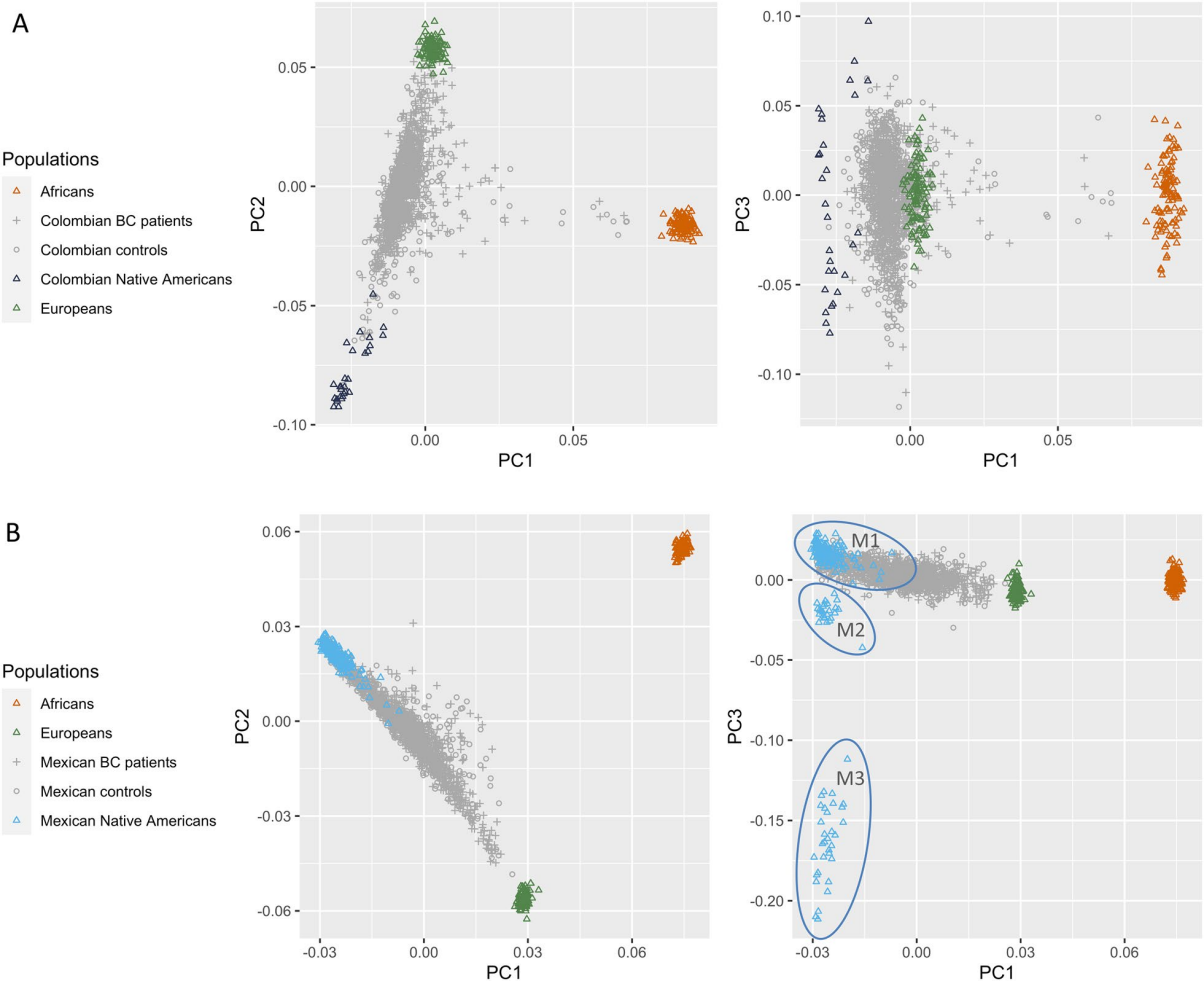
Scatter plots of first versus second, and first versus third genetic principal components (PC) of study participants (crosses: BC patients, circles: population-based controls) and reference panels of African, European and Native American ancestry (represented by triangles; M1 includes Maya, Mixe, Mixtec, Purepecha and Zapotec, M2 includes Tepehuano and Yaqui, and M3 includes Pima individuals); panel **A**: Colombian study, panel **B**: Mexican study

Figure 3 represents the first versus the second, and the first versus the third genetic principal components, along with the reference panels of Native American, European and African ancestry. The first three principal components explained a genetic variance of 4.07%, 1.34% and 0.18% in Colombian women and 5.89%, 2.00% and 0.26% for Mexican women, respectively. The first principal component distinguished between African and non-African ancestry in both admixed Colombian women (panel A) and admixed Mexican women (panel B). The second principal component distinguished between European and Native American ancestry. The third principal component separated three subtypes of Native American ancestry in Mexican women: the group of Native American reference individuals most similar to the study population (M1) included 115 individuals (49 Maya, 43 Zapotec, 17 Mixe, five Mixtec and one Purepecha).

**Fig. 4:**
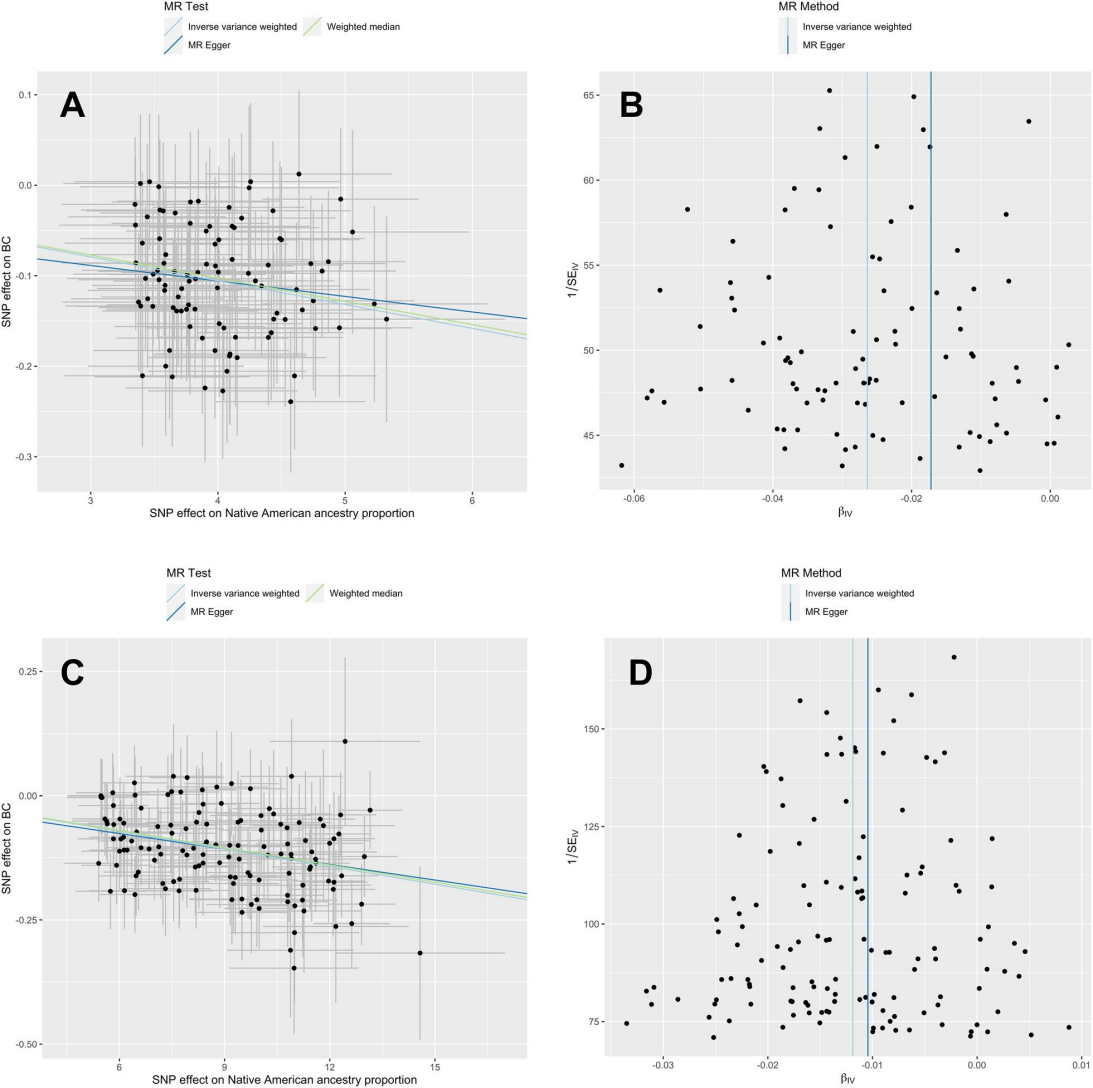
Scatter and funnel plots on the association between Native American ancestry and risk of breast cancer (**A** and **B**: Colombian study, **C** and **D**: Mexican study)

Table 1 shows the results for Colombian women, while the corresponding results based on Mexican women are summarised in Table 2. The 98 IVs utilised for two-sample MR in Colombian women explained a cumulative variance of 7.1% in the proportion of Native American ancestry (F-statistic = 55.57). The *p*-value of Cochran’s Q statistic (*p* > 0.99) revealed no instrument heterogeneity as an indicator of pleiotropy, the MR-Egger intercept (*p* = 0.60) was consistent with no horizontal pleiotropy, and departing instruments were not evident in scatter and funnel plots (Fig. 4, panels A and B), prompting the use of inverse variance-weighted (IVW) odds ratios (ORs) for the primary analyses. The cumulative variance explained by the 134 IVs used for the Mexican analyses amounted to 38.5% (F-statistic = 412.29), which is an atypically high proportion in Mendelian randomization studies. After inspection of the *p*-value of Cochran’s Q statistic (*p* > 0.99), the MR-Egger intercept (*p* = 0.66) and the scatter and funnel plots (Fig. 4, panels C and D), we also decided to use the IVW OR for the primary Mendelian randomization analyses in Mexican women.

**Table 1:** Results from main, sensitivity and stratified instrumental variable analyses on the association between Colombian Native American ancestry and breast cancer risk

	Method^1^	# IV	Exp. var.^2^ (%)	OR	95% CI		*p*-val	Q *p*-val^3^	intercept *p*-val^4^
*Main analysis*									
Two-sample MR	IVW	98	7.1	0.974	0.970	0.978	**3.1 × 10**^**–40**^	> 0.99	
*Sensitivity analyses*									
Two-sample MR	Egger	98	7.1	0.983	0.950	1.017	0.33	> 0.99	0.60
Two-sample MR	WM	98	7.1	0.975	0.969	0.980	**1.1 × 10**^**–20**^		
HGDP as reference^5^	IVW	95	6.6	0.974	0.970	0.978	**6.1 × 10**^**–37**^	> 0.99	
*Stratified analyses*									
BC diagnosed ≤ 45 y^6^	IVW	106	7.7	0.978	0.972	0.984	**1.2 × 10**^**–13**^	> 0.99	
Familial BC^7^	IVW	104	7.5	0.958	0.952	0.964	**2.0 × 10**^**–41**^	0.98	
ER-positive BC^8^	IVW	94	6.8	0.968	0.963	0.972	**7.1 × 10**^**–43**^	> 0.99	
ER-negative BC	IVW	111	8.1	0.988	0.981	0.995	**1.1 × 10**^**–03**^	> 0.99	
Triple-negative BC^9^	IVW	110	7.9	0.993	0.983	1.003	0.19	> 0.99	

Bold values indicate that the probability value is below 0.05

^1^IVW: Inverse variance weighted; Egger: MR-Egger regression; WM: weighted median estimates

^2^Exp. var.: Variance explained by the instrumental variables

^3^Cochran’s Q statistic *p*-values higher than 0.05 are suggestive of no instrument heterogeneity as a proxy for pleiotropy

^4^MR-Egger intercept *p*-values higher than 0.05 are consistent with no horizontal pleiotropy

^5^Individuals from the Human Genome Diversity Project were used instead of the 29 Native Americans from Colombia as the reference panel for estimation of global ancestry proportions

^6^Breast cancer diagnosed before the age of 45 years

^7^Breast cancer patients with first-degree relatives affected by breast and/or ovarian cancer

^8^Oestrogen receptor-positive breast cancer

^9^Oestrogen receptor-negative, progesterone receptor-negative and human epidermal growth factor receptor-negative breast cancer

**Table 2:** Results from main, sensitivity and stratified instrumental variable analyses on the association between Mexican Native American ancestry and breast cancer risk

	Method^1^	# IV	Exp. var.^2^ (%)	OR	95% CI		*p*-val	Q *p*-val^3^	intercept *p*-val^4^
*Main analysis*									
Two-sample MR	IVW	134	38.5	0.988	0.987	0.990	**1.4 × 10**^**–44**^	> 0.99	
*Sensitivity analyses*									
Two-sample MR	Egger	134	38.5	0.990	0.983	0.996	**2.6 × 10**^**–03**^	> 0.99	0.66
Two-sample MR	WM	134	38.5	0.988	0.986	0.991	**5.0 × 10**^**–22**^		
expl. Variance ~ 8%^5^	IVW	17	8.4	0.990	0.986	0.993	**6.7 × 10**^**–09**^	0.94	
HGDP as reference^6^	IVW	133	37.9	0.988	0.987	0.990	**7.4 × 10**^**–44**^	> 0.99	
refined Mexican reference^7^	IVW	134	38.6	0.988	0.987	0.990	**7.7 × 10**^–44^	> 0.99	
*Stratified analyses*									
BC diagnosed ≤ 45 y^8^	IVW	136	39.3	0.994	0.991	0.997	**1.4 × 10**^**–04**^	> 0.99	
Familial BC^9^	IVW	132	37.4	0.973	0.969	0.978	**1.0 × 10**^**–33**^	> 0.99	
ER-positive BC^10^	IVW	135	38.5	0.989	0.986	0.991	**6.6 × 10**^**–18**^	> 0.99	
ER-negative BC	IVW	139	40.2	1.004	1.001	1.008	**1.5 × 10**^**–02**^	> 0.99	
Triple-negative BC^11^	IVW	142	40.3	1.004	0.999	1.009	0.11	> 0.99	

Bold values indicate that the probability value is below 0.05

^1^IVW: Inverse variance weighted; Egger: MR-Egger regression; WM: weighted median estimates

^2^Exp. var.: Variance explained by the instrumental variables

^3^Cochran’s Q statistic *p*-values higher than 0.05 are suggestive of no instrument heterogeneity as a proxy for pleiotropy

^4^MR-Egger intercept *p*-values higher than 0.05 are consistent with no horizontal pleiotropy

^5^Subset of 17 IV explaining a cumulative variance in global proportion of Native American ancestry similar to the one in the Colombian study (8%)

^6^Individuals from the Human Genome Diversity Project were used instead of the 177 Native Americans from Mexico as the reference panel for estimation of global ancestry proportions

^7^The Maya, Mixe, Mixtec, Purepecha and Zapotec populations in the Human Genome Diversity Project were used as the reference panel for estimation of global ancestry proportions

^8^Breast cancer diagnosed before the age of 45 years

^9^Breast cancer patients with first-degree relatives affected by breast and/or ovarian cancer

^10^Oestrogen receptor-positive breast cancer

^11^Oestrogen receptor-negative, progesterone receptor-negative and human epidermal growth factor receptor-negative breast cancer

We found evidence of a putatively causal protective effect of Colombian Native American ancestry on BC risk (IVW OR = 0.974 per 1% increase in ancestry proportion, 95% confidence interval [CI] 0.970–0.978, *p* = 3.1 × 10^–40^). This estimate can also be interpreted as a 2.6% decrease in BC risk (95% CI 2.2–3%) per each 1% increase in the proportion of Native American ancestry (median 42% and interquartile range 36%-49% in Colombian female controls). MR results based on Mexican women also pointed to a protective effect of Native American ancestry on BC risk (IVW OR 0.988, 95% CI 0.987–0.990, *p* = 1.4 × 10^–44^). The median and interquartile range of the proportion of Native American ancestry were 65% and 54%-79% in Mexican control women, respectively.

Table 1 also shows the results of sensitivity analyses for Colombian women. The MR-Egger regression estimate (OR = 0.983, 95% CI 0.950–1.017) was consistent (overlapping 95% CI), and the weighted median estimate (OR = 0.975, 95% CI 0.969–0.980, *p* = 0.33) was virtually identical to the IVW OR (see also the regression lines on Fig. 4, panel A). To assess the influence on the results of the reference panel of Native American ancestry, we replaced the original 29 Native Americans from Colombia with 57 unrelated Native Americans from the HGDP (21 Maya, 13 Pima, 11 Karitiana, seven Piapoco and five Surui, see Additional file 1: Fig. 1) [31]. The use of Native Americans from the HGDP for calculation of ancestry proportions reduced the explained variance (from 7.1%-6.6%) and increased the *p*-value (to *p* = 6.1 × 10^–37^), but resulted in practically identical IVW OR estimates.

Stratified analyses in Colombian women suggested stronger protective effects of the proportion of Native American ancestry among women with a family history of BC and/or ovarian cancer (4.2% decrease in BC risk per 1% increase in proportion of Native American ancestry, 95% CI 3.6%-4.8%, *p* = 2.0 × 10^–41^), and on ER-positive BC (IVW OR = 0.968) versus ER-negative BC (IVW OR = 0.988) or triple-negative BC (IVW OR = 0.993; non-overlapping 95% CIs with ER-positive BC).

The results of the sensitivity analyses based on the Mexican women are shown in Table 2. As in the Colombian analyses, the IVW, MR-Egger and weighted median estimates were consistent (overlapping 95% CIs and very similar regression lines on Fig. 4, panel C). To examine the impact of the large variance explained by the IVs on the results, we reduced the number of IVs to 17, resulting in a similar explained variance as in the Colombian analyses (8.4%), but the IVW estimates barely changed (from OR = 0.988 to OR = 0.990). Neither the use of Native Americans from the HGDP nor the use of a subset of 115 Native Americans from Mexico (49 Maya, 43 Zapotec, 17 Mixe, five Mixtec and one Purepecha) as the reference panels for estimation of Native American ancestry proportions affected the IVW OR estimates.

Stratified analyses in Mexican women confirmed the stronger protective effect of Native American ancestry against familial BC found in Colombian women (2.7% decrease in familial BC risk compared with 1.2% decrease in overall BC risk per 1% increase in the proportion of Native American ancestry). Also in agreement with the Colombian results, the protective effect was stronger against ER-positive BC (IVW OR = 0.989) than against ER-negative BC (IVW OR = 1.004) or triple-negative BC (IVW OR = 1.004; non-overlapping 95% CIs and associated *p* < 2.2 × 10^–16^ with ER-positive BC).

Interestingly, most IVs (Colombian: 96%, Mexican: 97%) used as instrumental variables were population-specific, but differences between causal effect estimates based on common and population-specific markers did not reach statistical significance (overlapping 95% confidence intervals). For example, the IVW OR based on the four joint Colombian and Mexican IVs was 0.969 (95% CI 0.949–0.988) compared to 0.974 (95% CI 0.970–0.978) based on the 94 Colombia-specific IVs.

## Discussion

An inverse relationship between the individual proportion of Native American ancestry and BC risk has been previously reported in observational studies, but the role of confounding factors in this association remains unclear, and the standard approach of statistical adjustment is limited by (1) potentially large, non-random measurement errors in the adjustment variables (e.g. income and access to BC screening) and (2) the impossibility of taking into account as yet unknown risk factors. Using specific AIMs for Colombian and Mexican women as instrumental variables, we assessed the unconfounded effect of Native American ancestry on BC risk and found a confounder-free protective effect in both populations: each 1% increase in the proportion of Native American ancestry represented a 2.6% reduction in BC risk for Colombian women and a 1.2% risk reduction for Mexican women. Given the large variability in ancestry proportions in the populations investigated, these results are clinically relevant: they translate into expected differences in BC risk of (49–36) × 2.6 = 33.8% between Colombian women in the first and third ancestry quartiles—the corresponding BC risk difference for Mexican women was (79–54) × 1.2 = 30%. Extensive sensitivity analyses considering alternative reference panels of Native American ancestry, and different MR methods confirmed the protective association and its magnitude. Stratified analyses revealed stronger effects for familial BC, adding plausibility to our findings.

The OR estimated in this study for Colombian women (0.974 per 1% increase in Native American ancestry proportion, 95% CI 0.970–0.978) was similar to the OR adjusted for age, family history of BC, oral contraceptive use, menopausal status combined with postmenopausal hormone therapy use, body mass index, smoking status, parity, age at first full-term pregnancy and breastfeeding (OR = 0.976, 95% CI 0.969–0.983) previously reported in an observational study [23]. The study population investigated in the present study (714 BC patients and 687 matched female controls) largely overlapped with the population examined in the earlier observational study (722 BC patients and 622 matched female controls), which relied on targeted genotyping of 30 AIMs for ancestry estimation. As shown in Fig. 3, increasing proportions of Native American ancestry generally correspond to decreasing proportions of European ancestry in Latin American women, and European ancestry has previously been associated with increased BC risk among Mexicans and US Hispanics [21, 22]. Stratified analyses revealed a stronger protective effect of Native American ancestry against familial BC and ER-positive BC for both Colombian and Mexican women, which has important implications for BC prevention: As the negative association between Native American ancestry and BC risk previously found in observational studies appears to be free of confounding factors, taking into account the individual proportion of Native American ancestry could improve the effectiveness of current prevention strategies by, for example, defining the optimal ancestry-specific age for starting BC screening [32].

Based on 2107 BC cases and 2587 unaffected controls of Hispanic/Native American origin living in the United States and Mexico, Fejerman et al. examined the interaction between hormonal and lifestyle risk factors, and 10 BC susceptibility variants [33]. The authors found a correlation between Native American ancestry, hormone replacement use, and breastfeeding behaviour. They concluded that genetic ancestry in admixed populations may reflect not only population-specific differences in genetic susceptibility, but also differences in environmental exposures that, together with genetic factors, may influence BC risk. Hynes et al. also examined the interaction between established risk factors and genetic ancestry on BC risk among women from the USA and Mexico in the Breast Cancer Health Disparities Study [24]. They found that the inverse relationship between Native American ancestry and BC risk was only slightly attenuated when adjusted for known risk factors and concluded that established BC risk factors may be less relevant for women with a high proportion of Native American ancestry. Consistent with our findings, previous studies on the association between genetic ancestry and BC risk in Latin American populations have found differences in BC subtypes by ancestry. Rey-Vargas et al. reported a higher proportion of Native American ancestry in Colombian patients with ER+ /HER2+ (45%) than in ER + /HER2− (40%) breast tumours, and Marker et al. demonstrated a higher risk of HER2 + BC with increasing proportions of Native American ancestry in Colombian, Mexican and Peruvian women [34, 35]. The novelty of the present study lay in the use of instrumental variable analysis to identify and quantify the confounding-free effect of Native American ancestry on the risk of overall BC and BC subtypes.

The relatively low number of women investigated in the separate Colombian and Mexican analyses was a limitation of this study. On the other hand, the association found in previous observational studies was strong and the explained variance in the proportion of Native American ancestry was uncharacteristically high for instrumental variable analysis, especially in the Mexican substudy (38.5%), which translates into relatively high statistical power: Using the online tool provided by Brion et al., taking into account the available sample sizes and explained variances, and assuming a true OR of 0.72 per standard deviation of the proportion of Native American ancestry, as reported in the earlier study by Torres et al., the present study had a statistical power of 97% for the Mexican analyses (type I error rate of 5%) [23, 36]. Furthermore, sensitivity analyses for the Mexican substudy, in which we reduced the number of IVs to achieve an explained variance similar to the Colombian substudy, had little effect on the estimated ORs.

In addition to low statistical power, pleiotropy is an important limitation of MR studies. Since first-order inverse variance weights keep the type I error rate under the causal null, we calculated Cochran’s Q statistic using first-order weights to detect heterogeneity, which often reflects pleiotropy; visually inspected scatter and funnel plots; performed MR-Egger regression to detect potential bias attributable to horizontal pleiotropy; and used radial MR to detect potentially outlying IVs. The use of alternative reference panels of Native American ancestry to preselect ancestry informative markers in both Colombian and Mexican women, and the utilisation of a more specific reference panel in the Mexican analyses hardly influenced the estimated ORs (Table 2). The Colombians and Mexicans included in the less specific reference panels of Native American ancestry are genetically more similar to other Native Americans than to Europeans and Africans, but the general recommendation is to use reference panels that are genetically as close as possible to the study population.

The present findings, which support an unconfounded relationship between the proportion of Native American ancestry and BC risk—without the need for all BC risk factors to be known, accurately measured and statistically adjusted for—may pave the way for further admixture mapping studies that complement the successful identification of a novel BC susceptibility region on 6q25 by Fejermann et al., possibly in combination with subsequent association testing. Note that the genome-wide significance level for admixture mapping is much higher than for association mapping [37, 38]. From an implementation perspective, recruiting study women from regions with a high average proportion of Native American ancestry would increase the statistical power of admixture mapping.

In summary, in this first instrumental variable analysis of the confounding-free association between Native American ancestry and BC risk in genetically admixed Colombian and Mexican women, we found a protective unconfounded effect, which was stronger for familial and ER-positive BC. Confounder-free associations are generally more relevant than observational correlations, which could be attributed to unknown or known confounding factors that are potentially unmeasurable or systematically mismeasured, and the findings of the present study provide more refined information on the potential of accounting for ethnic differences (in this case, Native American ancestry) in disease prevention (in this case, BC). The methodology applied in this investigation can be used to distinguish between confounded and unconfounded effects of genetic ancestry on other disease outcomes, contributing to understand and hopefully also reduce health disparities, and also to obtain more accurate information for future admixture mapping studies. From a more applied point of view, the present results provide strong evidence for a putatively causal effect of the proportion of Native American ancestry on BC risk, which may have direct implications for the design of more effective BC prevention strategies for Latin American women and Latinas.

## Material and methods

### Study populations

We used individual-level data from two previous studies contributing to the Breast Cancer Association Consortium (BCAC): The Colombian Breast Cancer Case–Control (COL-BCCC) study as described in [23, 39] and the Mexican Cancér de Mama (CAMA) study as described in [22, 40–42]. In brief, Colombian cases were women with a BC diagnosis after 01/01/2004 recruited between 03/2007 and 02/2011 in hospitals from Bogota, Neiva and Villavicencio, which mainly attend women from the central Andean regions of Cundinamarca and Huila. Controls were healthy, unrelated women from the same regions who participated in the Colombian National Pap smear programme and did not report a family history of breast cancer or any other type of cancer in two generations. Mexican BC patients were recruited between 01/2004 and 12/2007 in hospitals from Mexico City, Monterrey and Veracruz. Eligible cases were women with a new BC diagnosis without previous treatment who were not pregnant. Controls were matched to cases according to 5-year age groups, membership to health care institution and place of residence. In both studies, additional information was collected using health questionnaires and genomic DNA was extracted from peripheral blood. Genotypes in BCAC were determined using the Illumina custom array OncoArray [43]. Genotype and phenotype data were available from 687 Colombian BC patients (and 714 matched female controls) and from 709 Mexican BC patients (and 657 matched female controls).

The following reference panels of individuals of Native American ancestry were used for preselection of ancestry-informative markers: (1) primary Colombian analyses: 29 Colombian Native Americans (nine Inga, five Arhuaco, four Embera, three Kogi and three Waunana described by Reich et al. [30] and five Piapoco individuals from the HGDP [31]); (2) primary Mexican analyses: 177 Mexican Native Americans (49 Maya, 43 Zapotec, 30 Pima, 25 Tepehuano, 17 Mixe, five Mixtec, one Yaqui and one Purepecha described by Reich et al. [30] and four Maya and one Pima from the HGDP [31]); (3) secondary analyses, Colombian and Mexican I: 57 Native Americans from the HGDP [31] (seven Piapoco, 11 Karitiana, 21 Maya, 13 Pima, five Surui; and (4) secondary analyses, Mexican II: 115 Mexican Native Americans (49 Maya, 43 Zapotec, 17 Mixe, 5 Mixtec and one Purepecha) described by Reich et al. [30].

## Methods

The study was performed in parallel in a Colombian and a Mexican study population. For each population independently, we first preselected ancestry-informative markers (AIMs) as instrumental variables (IVs) to assess the confounding-free association between the proportion of Native American ancestry and BC risk. Further sensitivity analyses and stratified analyses by characteristics in BC patients completed this study.

Mendelian randomization (MR) relies on the random assortment of genetic variants during meiosis, yielding a random distribution of alleles in the study population. Given the parental genotypes in an admixed population, the alleles of AIMs are randomly assigned at meiosis, mimicking a trial randomized by nature. We therefore first preselected genetic variants by using a method to determine the amount of information that multiallelic variants provide about individual ancestry composition, the so-called informativeness for assignment measure* I*_*n*_ [28]. For each genetic variant with *j* = 1, 2 possible alleles and for *i* = 1,…,*K* subpopulations we calculated:

$$I_{n} \left( {Q,J} \right) = \mathop \sum \limits_{j = 1}^{2} \left( { - p_{j} \log \left( {p_{j} } \right) + \mathop \sum \limits_{i = 1}^{K} \frac{{p_{ij} }}{K}\log \left( {p_{ij} } \right)} \right),$$

where *p*_*j*_ denotes the average frequency of allele *j* in all *K* subpopulations, *p*_*ij*_ the average frequency of allele *j* in subpopulation *i*, *Q* the (random) assignment of an individual to a subpopulation, and *J* the (random) genotype of one of the two alleles of an individual. The higher the *I*_*n*_-value the more information about ancestry composition is provided by a genetic variant. We used the reference panels described above to calculate the *I*_*n*_*-*value considering two populations at a time (Native American and European, Native American and African). We then preselected the 20,000 genetic variants with the highest *I*_*n*_*-*value for each comparison and retained the variants that were present in at least one comparison as preselected markers of Native American ancestry.

To select valid IVs among the preselected AIMs, we selected genetic variants that were (1) robustly associated with the investigated exposure (individual proportion of Native American ancestry), (2) not associated with potential confounders, and (3) in linkage equilibrium with one another. To fulfil (1), we used the software ADMIXTURE, version 1.3.0, for supervised estimation of individual proportions of Native American ancestry using the reference panels described above [44] and retained preselected markers with a probability value for the association with the proportion of Native American ancestry lower than 5 × 10^–8^. To fulfil (2), we performed a PheWAS and excluded markers associated with the following phenotypes provided by MR Base: menopause, pregnancy outcomes, tobacco or cigarette smoking, alcohol consumption, educational level, contraceptives, hormone-replacement therapy, diabetes, body circumferences and breast cancer [45]. To fulfil (3), we calculated the explained variance in the proportion of Native American ancestry using the formula ^36^:

$${\text{explained}}\;{\text{variance}} = \beta^{2} \times 2 \times {\text{MAF}} \times \left( {1 - {\text{MAF}}} \right),$$

where *β* denotes the additive genetic effect and MAF denotes the allele frequency. For markers in linkage disequilibrium, which we separately identified in the 714 Colombian and the 657 Mexican female controls using the R package ‘genetics’ [46], we retained the marker with the highest explained variance. The cumulative variance explained was then calculated by adding the variances explained by all retained markers.

To visually inspect and subsequently exclude outlying IVs in the main analyses, as well as in the sensitivity and stratified analyses, we first used summary statistics adjusted for age of the association between the IVs and the exposure of interest (Native American ancestry proportion), and of the association between the IVs and the investigated outcome (BC), based on the control females as sample 1, and the BC patients and matched control females as sample 2. After performing radial MR using the R package ‘RadialMR’ [47], non-excluded markers were used as IVs to perform two-sample MR analyses using the R-version of MR-Base [45]. We also calculated Cochran’s Q statistic using inverse variance weights to preclude heterogeneity, which may indicate violation of the IV assumptions.

To complement the main results, we performed sensitivity analyses and stratified analyses. Sensitivity analyses included the calculation of weighted median estimates and MR-Egger regression to test for directional pleiotropy. To examine the possible impact of less specific reference panels, we reran the analyses with ancestry proportions estimated by replacing the primary reference individuals for Colombian and Mexican Native American ancestry with reference individuals for general Native American ancestry. The influence of a more specific reference panel was assessed by visually inspecting the principal component plots of the Mexican study population and then re-estimating the ancestry proportions only with those reference individuals genetically most similar to the study Mexican BC patients and matched controls. Genetic principal components were estimated for Colombian and Mexican study participants separately, using the respective reference panels and the eigenstrat function [48]. Additionally, we repeated the analyses limiting the number of IVs, so that the explained variance in the Mexican study was similar to the explained variance in the Colombian study. Finally, we performed stratified analyses considering only patients diagnosed before the age of 46 years, patients with a family history of breast and/or ovarian cancer, and patients affected by oestrogen-receptor (ER) positive or negative tumours, or by triple-negative BC (negative for ER, progesterone receptor, and human epidermal growth factor receptor 2).

### Supplementary Information

**Additional file 1: Fig. 1.** Scatter plots of first versus second, and first versus third genetic principal components (PC) of study participants (crosses: BC patients, circles: population-based controls) and reference panels of African, European and Native American ancestry (reference individuals represented by triangles; Native Americans from the Human Genome Diversity Project, N1: Karitiana, N2: Surui, N3: Piapoco, N4: Maya, N5: Pima); panel A: Colombian study, panel B: Mexican study.

## Data Availability

The source code to reproduce all the results and the necessary input files with the summary association statistics are available at www.biometrie.uni-heidelberg.de/StatisticalGenetics/Software_and_Data.
