# Supplementary material for: Native American ancestry and breast cancer risk in Colombian and Mexican women: ruling out potential confounding through ancestry-informative markers
Source: Breast Cancer Res. 2023 Oct 2;25:111. doi: 10.1186/s13058-023-01713-5 (PMC10544431; doi:10.1186/s13058-023-01713-5)
Supplement: Supplementary file 1 — Additional file 1: Fig. 1. Scatter plots of first versus second, and first versus third genetic principal components (PC) of study participants (crosses: BC patients, circles: population-based controls) and reference panels of African, European and Native American ancestry (reference individuals represented by triangles; Native Americans from the Human Genome Diversity Project, N1: Karitiana, N2: Surui, N3: Piapoco, N4: Maya, N5: Pima); panel A: Colombian study, panel B: Mexican study. [file 13058_2023_1713_MOESM1_ESM.docx]

| **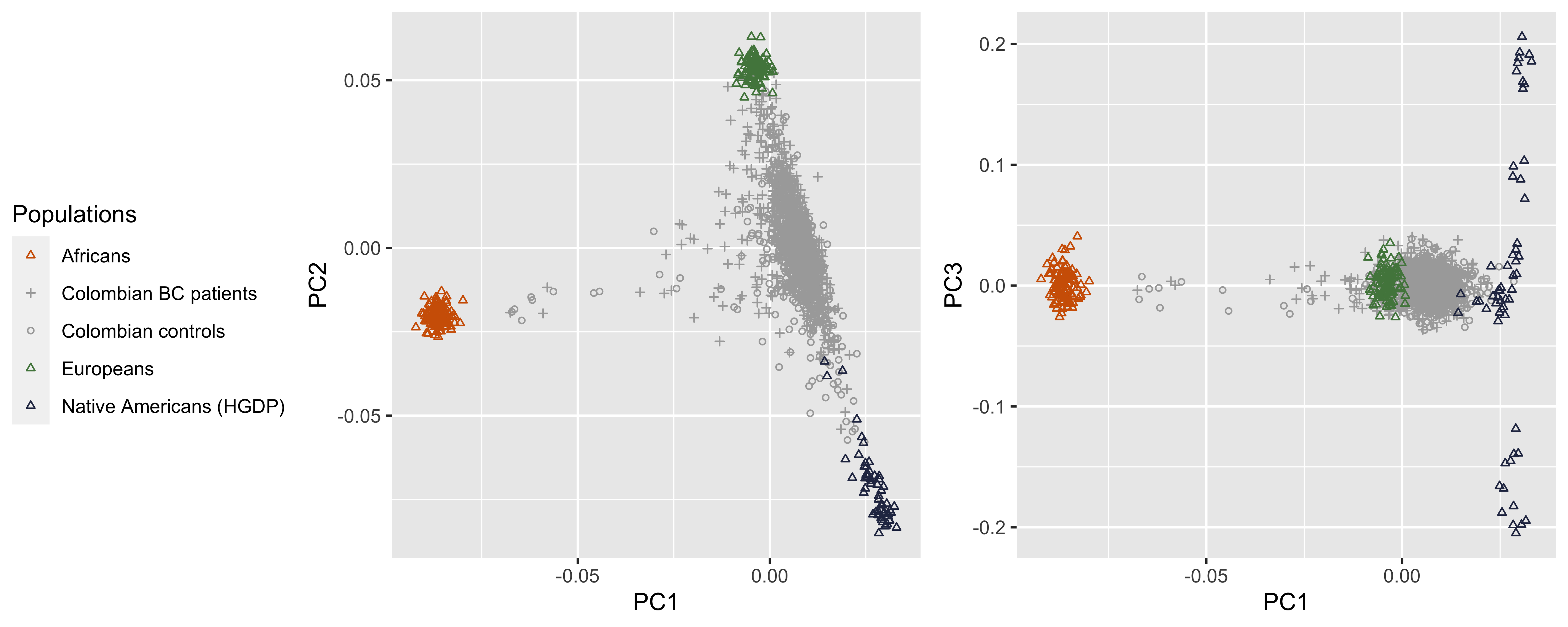**  N5  N4  N3  N1  N4  N3  N2  N1  A |
| --- |
| **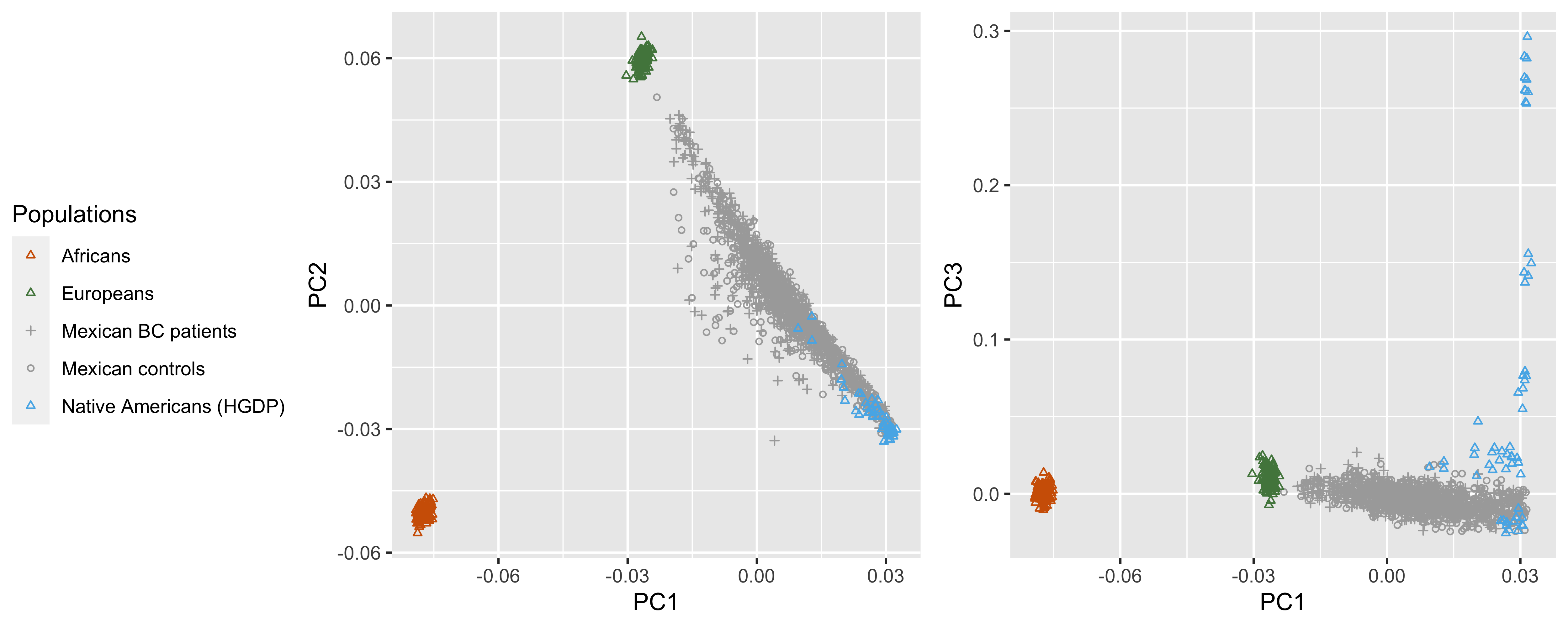**  N2  N5  B |

**Supplementary Figure 1:** Scatter plots of first versus second, and first versus third genetic principal components (PC) of study participants (crosses: BC patients, circles: population-based controls) and reference panels of African, European and Native American ancestry (reference individuals represented by triangles; Native Americans from the Human Genome Diversity Project, N1: Karitiana, N2: Surui, N3: Piapoco, N4: Maya, N5: Pima); panel A: Colombian study, panel B: Mexican study
